# Association of a Low Eosinophil-to-Monocyte Ratio With One-Month All-Cause Mortality in Patients With ST-Segment Elevation Myocardial Infarction: A Cohort Study

**DOI:** 10.7759/cureus.114968

**Published:** 2026-08-22

**Authors:** Somia Pervaiz, Zunaira Javed, Aqeel Asghar, Ayesha Khaqan, Syed M Abubakar Jilani, Ali Hameed, Shabbar H Changazi

**Affiliations:** 1 Cardiology, Mayo Hospital, Lahore, PAK; 2 Cardiology, Punjab Institute of Cardiology, Lahore, PAK; 3 Cardiology, King Fahad Medical City, Riyadh, SAU; 4 Cardiology, CMH Lahore Medical College and Institute of Dentistry, Lahore, PAK; 5 General Surgery, Provincial Headquarter Teaching Hospital, Gilgit, PAK

**Keywords:** cardiovascular mortality, eosinophil-to-monocyte ratio, inflammatory biomarkers, low-resource settings, risk stratification, stemi

## Abstract

Introduction

ST-elevation myocardial infarction (STEMI) remains a major driver of premature cardiovascular death across South Asia, where many hospitals must triage patients without routine access to advanced risk-stratification tools. The eosinophil-to-monocyte ratio (EMR), derived at no added cost from the complete blood count (CBC) every STEMI patient already receives, has been proposed as a low-cost prognostic marker. This study examined whether EMR at admission is independently associated with one-month all-cause mortality among STEMI patients in Pakistan.

Methods

This prospective cohort study enrolled 226 patients aged 18-50 years with confirmed STEMI at the Emergency Department of the Punjab Institute of Cardiology, Lahore, Pakistan, from August 20, 2020, to February 20, 2024: 113 with a low admission EMR (<0.18) and 113 without (≥0.18). Patients were followed for one month for all-cause mortality, and independent predictors of mortality were identified using multivariate logistic regression adjusted for age, sex, hypertension, diabetes, smoking, and previous myocardial infarction.

Results

Among the 226 patients followed for 30 days, those with a low EMR died at more than three times the rate of those without (9.7% versus 2.7%, p = 0.027), and low EMR remained an independent predictor of death after adjustment for traditional risk factors using Firth's penalized logistic regression (adjusted odds ratio (AOR) 3.89, 95% confidence interval (CI) 1.21-13.84, p=0.025).

Conclusion

In this cohort of STEMI patients, a low EMR at admission was independently associated with significantly increased mortality at one month (AOR 3.89; 95% CI 1.21-13.84; Firth's penalized logistic regression). Because the confidence interval is wide and the number of deaths is small, these results are hypothesis-generating, and a larger multi-center, external validation study is required before EMR can be recommended for STEMI triage protocols. If these results are replicated, the benefits of implementing EMR could be compelling for risk stratification in health systems where cutting-edge diagnostics are not widely adopted due to its close-to-zero marginal cost.

## Introduction

Myocardial infarction is not evenly distributed in its consequences. A patient who develops ST-elevation myocardial infarction (STEMI) in a well-resourced urban center with round-the-clock catheterization capability faces very different odds than one who develops the same disease two or three hours from the nearest cath lab [1]. Pakistan, like much of South Asia, carries a heavy and rising burden of coronary artery disease, and STEMI patients here are frequently risk-stratified with little more than an electrocardiogram, a basic troponin, and clinical judgment, even though early risk stratification is considered a cornerstone of routine STEMI care [2]. Established prognostic tools for this purpose, such as the GRACE and TIMI risk scores and serial high-sensitivity troponin measurement, remain difficult to implement in most public-sector hospitals in the region, largely because of the infrastructure, laboratory turnaround time, and training they require rather than because of clinical need, which is why the search for simple, inexpensive prognostic markers continues [3].

Inflammation is central to how STEMI progresses and how patients fare afterward. Neutrophils, lymphocytes, and monocytes all participate in plaque rupture, vascular dysfunction, and post-infarct left ventricular remodeling [4], and ratios built from these cells, such as the neutrophil-to-lymphocyte ratio and the lymphocyte-to-monocyte ratio, have shown real predictive value in prior work [1,5]. Their performance, however, is not always consistent across populations [6], and none of them cost anything beyond a complete blood count (CBC) that is already drawn on essentially every patient who walks into an emergency department with chest pain.

Eosinophils add a further layer to this picture. They appear to help regulate the post-infarct inflammatory response by modulating platelet adhesion and releasing immunosuppressive cytokines [7,8], and a single prior study by Deng et al. in China found that a low eosinophil-to-monocyte ratio (EMR) on admission predicted both short- and long-term mortality in STEMI patients treated with primary percutaneous coronary intervention (PCI) [1]. Whether that relationship holds in other populations and other health-system contexts, including ones where primary PCI is not universally available, has not been established.

This study was designed to examine whether there is an independent association between EMR at admission and one-month all-cause mortality among patients with STEMI who underwent treatment at a tertiary cardiac center in Pakistan after adjusting for traditional risk factors. This analysis is intended to generate evidence on whether EMR merits further investigation as a potential low-cost prognostic marker. We do not claim to validate EMR for clinical use, but rather to assess whether the association observed in previous studies can be replicated in a different health-system context.

## Materials and methods

This was a prospective cohort study conducted in the Emergency Department of the Punjab Institute of Cardiology, Lahore, Pakistan, from August 20, 2020, to February 20, 2024. Patients between 18 and 50 years old who presented to the Emergency Department with a confirmed STEMI were screened consecutively and were separated in the Emergency Department into non-exposed (EMR ≥0.18) and exposed (EMR <0.18) groups based on Emergency Department EMR status. The sample size was determined a priori with 7.09% and 0.7% expected one-month mortality rates in the exposed and non-exposed groups, respectively, reported by Deng et al., which resulted in a target of 113 patients per exposure group. Recruitment was carried out sequentially within each exposure stratum until the pre-specified target of 113 was achieved in each group (226 enrolled total). This was not simple consecutive sampling but a non-probability, exposure-stratified consecutive sampling procedure: the sizes of the last two groups were determined a priori by the power calculation, and the groups were not strictly matched as 1:1 matched pairs but rather each group was accrued consecutively and independently until the target size was achieved. Exclusion criteria included history of cancer, active infection, rheumatic or allergic disease, congenital or valvular heart disease, cardiomyopathy, STEMI secondary to substance abuse, presentation more than 48 hours after symptom onset, death within 24 hours of admission, or coronary thrombus occurring from atrial fibrillation. No patient data were missing for the exposure, outcome, or covariates analyzed, so no imputation was required.

After informed consent, demographic and clinical history, including hypertension, diabetes, smoking, and previous myocardial infarction, were recorded on a structured proforma. A blood sample drawn at admission was analyzed for CBC, cardiac troponin T, serum creatinine, and C-reactive protein. EMR was calculated as the absolute eosinophil count divided by the absolute monocyte count. Patients were followed for one month by telephone contact or hospital-record review to ascertain all-cause mortality.

Data were analyzed in IBM SPSS Statistics for Windows, Version 25.0 (Released 2017; IBM Corp., Armonk, NY, USA). Categorical variables were summarized as frequencies and percentages and compared with the chi-square test; continuous variables were summarized as mean ± standard deviation. The number of events (14) and the number of covariates (7) resulted in a ratio of events per variable of approximately 2:1, which is below the standard of 10 events per variable for primary multivariable modeling; Firth's penalized logistic regression was used to fit the main multivariable model, which is a modification of logistic regression to reduce small-sample bias in sparse-data logistic regression, and profile penalized-likelihood 95% confidence intervals (CIs) are reported. A conventional (non-penalized) maximum-likelihood logistic regression model using the same covariates was also fit for comparison. Low EMR, age, sex, hypertension, diabetes, smoking, and previous myocardial infarction were included as covariates in both models, and the adjusted odds ratios (AORs) with 95% CI are presented. A two-sided p-value of ≤0.05 was deemed statistically significant.

The study was approved by the Institutional Review Board of the Punjab Institute of Cardiology, Lahore (reference number: RTPGME-Research-126A). Written informed consent was obtained from all participants, and patient confidentiality was maintained throughout.

## Results

Baseline characteristics were well balanced between the two groups. Mean age was 37.9 ± 9.3 years in the low-EMR group versus 39.5 ± 8.2 years in the non-low-EMR group (p=0.17), and the distribution of sex, hypertension, diabetes, smoking, and prior MI did not differ significantly between groups (Table 1).

**Table 1:** Baseline Characteristics of STEMI Patients by EMR Group The test statistic is the Pearson chi-square (χ², df=1) for categorical variables and the independent-samples t-test (t, df=224) for age; no separate statistic is given for the female row, which is the complement of the male row. SD, standard deviation; MI, myocardial infarction; STEMI, ST-elevation myocardial infarction; EMR, eosinophil-to-monocyte ratio

Characteristic	Low EMR (n=113)	Non-low EMR (n=113)	p-value	Test statistic
Age, years, mean ± SD	37.9 ± 9.3	39.5 ± 8.2	0.17	t=1.37
Male, n (%)	66 (58.4)	71 (62.8)	0.50	χ²=0.46
Female, n (%)	47 (41.6)	42 (37.2)	-	-
Hypertension, n (%)	37 (32.7)	38 (33.6)	0.89	χ²=0.02
Diabetes mellitus, n (%)	47 (41.6)	49 (43.4)	0.79	χ²=0.07
Smoking, n (%)	35 (31.0)	38 (33.6)	0.67	χ²=0.18
Previous MI, n (%)	12 (10.6)	16 (14.2)	0.42	χ²=0.65

One-month all-cause mortality was significantly higher in the low-EMR group: 11 of 113 patients (9.7%) died, versus 3 of 113 (2.7%) in the non-low-EMR group (p=0.027) (Table 2).

**Table 2:** One-Month All-Cause Mortality by EMR Group p-value and test statistic (Pearson chi-square, χ², df=1) compare the proportion of deaths within one month between the low-EMR and non-low-EMR groups; no separate statistic is given for the survived row, which is the complement of the mortality row. EMR, eosinophil-to-monocyte ratio

Mortality	Low EMR (n=113)	Non-low EMR (n=113)	Total	p-value	Test statistic
Died within 1 month, n (%)	11 (9.7)	3 (2.7)	14 (6.2)	0.027	χ²=4.87
Survived, n (%)	102 (90.3)	110 (97.3)	212 (93.8)	-	-

After adjustment for age, sex, hypertension, diabetes, smoking, and previous MI using Firth's penalized logistic regression, low EMR remained the only variable independently associated with one-month mortality, with almost four times the adjusted odds of death (AOR 3.89, 95% CI 1.21-13.84, p=0.025) (Table 3). No other covariate reached statistical significance in the adjusted model. Results were consistent with the conventional (non-penalized) logistic regression model, which produced a similar but less precise estimate (AOR 4.12, 95% CI 1.09-15.58, p=0.037), supporting the robustness of the association despite the low number of events.

**Table 3:** Multivariate Firth's Penalized Logistic Regression for Predictors of One-Month Mortality Firth's penalized logistic regression was used to address the low events-per-variable ratio (14 deaths/7 covariates = 2:1); profile penalized-likelihood confidence intervals are reported. OR, odds ratio; CI, confidence interval; MI, myocardial infarction; EMR, eosinophil-to-monocyte ratio

Variable	Adjusted OR	95% CI	p-value
Low EMR (vs. non-low)	3.89	1.21-13.84	0.025
Age, per year	1.00	0.94-1.08	0.95
Male sex (vs. female)	1.87	0.44-8.21	0.39
Hypertension	2.68	0.74-10.72	0.13
Diabetes mellitus	2.18	0.60-8.54	0.23
Smoking	2.41	0.65-9.48	0.19
Previous MI	1.09	0.20-5.83	0.92

## Discussion

The headline finding is a practical one: a ratio calculated for free from a test almost every STEMI patient already receives identified in this cohort which patients were most likely to die within a month. That is worth taking seriously in a health system where the alternative risk-stratification tools are often unavailable, unaffordable, or both.

These results align closely with the earlier report by Deng et al., who found that low EMR independently predicted both short- and long-term mortality in STEMI patients undergoing primary PCI in China [1]. The mortality rates observed here, 9.7% versus 2.7%, are broadly consistent with their 7.09% versus 0.7%, and this study extends the association to a younger South Asian cohort treated in a setting where primary PCI access is less uniform. Mechanistically, a low EMR likely reflects a combination of relative monocytosis, reflecting monocyte recruitment into the damaged myocardium and atherosclerotic plaque [9-11], and relative eosinopenia, which may result from eosinophils promoting platelet adhesion and infiltrating the coronary thrombus while releasing immunosuppressive cytokines such as IL-4, IL-10, and IL-13 [12,13]. This eosinopenia likely reflects both the migration of eosinophils out of peripheral blood and into the ruptured plaque and thrombus [14] and a stress-driven rise in cortisol that suppresses circulating eosinophil counts [15].

For health systems in Pakistan and comparable settings, the practical appeal of EMR is that it requires no new equipment, no new test, and no new training beyond reading a number that is already sitting in the CBC report. While awaiting external validation in larger, multi-center cohorts, EMR is a candidate for prospective evaluation as an adjunct to the STEMI triage protocols in district and tertiary hospitals to help emergency physicians assess, when a patient arrives in their hospital, whether the patient is likely to require further detailed investigations, prompt specialist involvement, or transfer to a PCI-capable hospital.

Realizing that potential would require more than a positive study, however. The provincial health departments and hospital administration would have to determine if this should be formally integrated into the EMR workflow in the Emergency Department, which would involve educating clinicians, implementing a process to monitor the performance of the CBC turnaround time, and defining what would happen if this EMR result is low. There is also a real trade-off: if using the EMR in this study resulted in an unnecessary escalation of care for some patients (false alarm) or if clinicians relied solely on this imperfect marker rather than known risk factors, then this could lead to complacency for others. The use of EMR should not be independent of but rather a supplement to the traditional STEMI risk assessment.

The principal strength of this study is its focus on an index that costs nothing beyond a test already performed. The conventional (non-penalized) adjusted effect estimate for low EMR had a wide 95% CI (1.09-15.58), reflecting the sparse-data setting created by only 14 deaths across 226 patients and seven covariates (approximately two events per variable, well below the commonly cited threshold of 10). To address this, the primary multivariable model was re-fit using Firth's penalized logistic regression, which produced a materially similar but more precise adjusted association (AOR 3.89, 95% CI 1.21-13.84, p=0.025), supporting the robustness of the EMR-mortality association despite the limited number of events; the result should nonetheless still be regarded as hypothesis-generating given the modest overall sample size and event count. The role of serial EMR measurement was not evaluated, so whether serial measurement would clarify its prognostic role remains unknown. This study did not capture door-to-balloon time, infarct size, or reperfusion strategy, so residual confounding from these unmeasured factors is possible and could not be adjusted for. The single-center design and the non-probability, exposure-stratified sampling strategy limit generalizability to broader STEMI populations. The cohort was also limited to patients aged 18-50, and these results might not apply to older patients, who represent the larger proportion of STEMI patients globally.

Larger, multi-center studies across a broader age range, with longer follow-up, are needed before EMR thresholds are written into clinical protocols. Studies that pair EMR with cost-effectiveness or budget-impact analysis would help health departments weigh its adoption against competing priorities for scarce cardiac-care funding.

## Conclusions

In this cohort of STEMI patients in Pakistan, a low EMR at admission was independently associated with substantially higher one-month all-cause mortality (AOR 3.89, 95% CI 1.21-13.84) using Firth's penalized logistic regression, a method that provides more reliable estimates in rare-event data. This association is consistent with findings from prior studies and supports the hypothesis that EMR may have prognostic value. However, because the confidence intervals are wide and the number of deaths is small, these results should be considered hypothesis-generating. Larger, multi-center studies with external validation are required before EMR can be recommended for incorporation into clinical triage protocols. Should these findings be confirmed, the fact that EMR is derived at no additional cost from a routine test could make it an attractive candidate for low-resource settings where advanced risk-stratification tools remain scarce.
